# Case Report: First occurrence of smoldering multiple myeloma in activated phosphoinositide 3-Kinase δ syndrome

**DOI:** 10.3389/fonc.2026.1859289

**Published:** 2026-06-11

**Authors:** Yanqiu Li, Linlin Huang, Wenhua He, Xin Wang, Ningning Tang, Dongmei Zhu, Xingli Jia, Chao Ding

**Affiliations:** 1 Department of Hematology, Suining Central Hospital, Suining, Sichuan, China; 2 Department of Hematopathology, Sichuan Kingmed Center for Clinical Laboratory, Chengdu, Sichuan, China

**Keywords:** activated phosphoinositide 3-kinase δ syndrome, case report, PIK3CD gene, recurrent infection, smoldering multiple myeloma

## Abstract

Activated phosphoinositide 3-kinase δ syndrome (APDS) is a newly described inborn error of immunity. Heterozygous mutations in the PI3Kδ catalytic subunit p110δ *(PIK3CD*) or regulatory subunit p85α (*PIK3R1*) lead to APDS1 and APDS2, respectively. Clinical manifestations include lymphoproliferation, recurrent respiratory tract infections, herpesvirus infections, enteropathy, elevated IgM, autoimmunity, and increased susceptibility to tumors, particularly lymphoma. Smoldering multiple myeloma (SMM), an asymptomatic clonal plasma cell disorder considered a pre-malignant entity, may progress to symptomatic multiple myeloma (MM) over time. This report describes a young patient with recurrent respiratory tract infections (pneumonia, bronchiectasis, and otitis media), lymphoproliferation, genital herpes, autoimmunity, and elevated serum IgM and IgG levels. Whole-exome sequencing identified a heterozygous *PIK3CD* gene mutation (c.1002C>G, p.Asn334Lys), and Sanger sequencing confirmed the same mutation in the patient’s son. Bone marrow aspiration and serum immunofixation electrophoresis confirmed monoclonal plasma cell proliferation, supporting a diagnosis of SMM. The patient did not receive targeted therapy. Early genetic sequencing can facilitate timely diagnosis of APDS and improve prognosis. To the best of our knowledge, this is the first reported case of APDS coexisting with SMM.

## Introduction

Activated phosphoinositide 3-kinase δ syndrome (APDS) is a primary immunodeficiency disorder first reported in 2013 (1). It is an autosomal dominant condition with an estimated prevalence of approximately 1 in 1 million (2). The phosphoinositide 3-kinase (PI3K) complex comprises a catalytic subunit (p110α, p110β, or p110δ) and a regulatory subunit (p85α, p55α, p50α, p85β, or p55γ). Gain-of-function variants in *PIK3CD* (encoding p110δ) cause APDS1, while loss-of-function variants in *PIK3R1* (encoding p85α) lead to APDS2. Disease severity and clinical trajectories vary widely among patients. Typical clinical features of APDS include recurrent sinopulmonary infections, gastrointestinal disorders, autoimmunity, non-malignant lymphoproliferation, lymphomas, and impaired control of Epstein-Barr virus (EBV), cytomegalovirus (CMV), and varicella-zoster virus (VZV) infections (3–5). The most common APDS1-associated mutation is *PIK3CD* c.3061G>A (p.E1021K) (6), whereas the *PIK3CD* c.1002C>G (p. Asn334Lys) variant has been documented in only one previous case, presenting as recurrent panuveitis (7). APDS patients have a ~13% risk of developing lymphoma, including diffuse large B-cell lymphoma, Hodgkin lymphoma, and marginal zone B-cell lymphoma (8). Current therapeutic strategies for APDS include immunoglobulin replacement therapy, antibiotic prophylaxis, mammalian target of rapamycin (mTOR) inhibitors, and hematopoietic stem cell transplantation (HSCT) (9). In March 2023, leniolisib—a novel, orally bioavailable small-molecule inhibitor targeting PI3Kδ signaling—was approved by the U.S. Food and Drug Administration (FDA) for APDS treatment (10). Smoldering multiple myeloma (SMM) is a pre-malignant plasma cell disorder that may progress to symptomatic diseases such as multiple myeloma (MM) or AL amyloidosis. The annual risk of progression from SMM to MM is ~10% in the first five years, decreasing to 3% thereafter (11).

This report presents an extremely rare case of APDS concurrent with SMM, expanding the known tumor spectrum associated with APDS and providing insights into potential pathogenic links between PI3K signaling dysregulation and plasma cell disorders.

## Case description

The female patient was born in December 2000 and had a history of recurrent cough and fever since childhood, which were attributed to respiratory tract infections. In 2016, she was admitted to the nephrology department due to bilateral lower extremity edema. A complete blood count showed a white blood cell count of 5.2 × 10^9^/L (normal range [NR], 3.5–9.5× 10^9^/L), hemoglobin of 139 g/L (NR, 110–150 g/L), and platelet count of 89 × 10^9^/L (NR, 100–300 × 10^9^/L). The direct Coombs test was positive (+ +), while the indirect Coombs test was negative. Biochemical tests indicated an albumin level of 37.9 g/L (NR, 40–55 g/L), with normal globulin, liver enzymes (ALT, AST), bilirubin, and renal function. Immunoglobulin and complement levels revealed elevated IgM (8.97 g/L; NR, 0.63–2.77 g/L) and decreased C3 (0.36 g/L; NR, 0.85–1.93 g/L) and C4 (0.05 g/L; NR, 0.12–0.35 g/L). Antinuclear antibody (ANA) profiling showed a titer of 1:1000 with a speckled pattern, along with positive results for anti-SSA (subtype not specified by the laboratory), positive anti-Ro52, and positive anti-SSB; anti-Ro60 was not tested. Urinalysis indicated proteinuria (quantitative protein: +2). She was diagnosed with systemic lupus erythematosus (SLE) and treated with prednisone acetate combined with hydroxychloroquine, which she later discontinued on her own initiative due to subjective improvement and side effects (weight gain, gastrointestinal discomfort). Since 2017, the patient has been repeatedly hospitalized in the Department of Respiratory Medicine due to recurrent cough and sputum production. Sputum cultures confirmed the presence of *Pseudomonas aeruginosa* and *Proteus penneri*, and computed tomography (CT) scans revealed bronchiectasis with concurrent infection (**Figure 1**).

**Figure 1:**
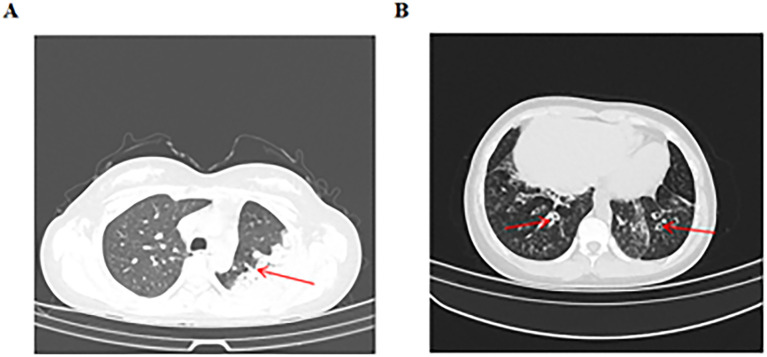
**(A)** CT scan showed exudative lesions in the left lower lobe of the lung(Red arrow). **(B)** CT scan revealed multiple bronchiectasis in both lungs(Red arrow).

In July 2020, she underwent a cesarean section due to fetal distress and failure to progress and delivered a male infant. In November 2020, she presented to the otolaryngology department with bilateral otorrhea and hearing loss; surgical intervention confirmed a diagnosis of acute suppurative otitis media with cholesteatoma. Since 2020, she has also experienced bilateral epiphora and increased discharge and was subsequently diagnosed with bilateral chronic dacryocystitis. Surgical treatment was administered, but postoperative tear duct irrigation remained obstructed with purulent discharge. During the same period, she suffered from recurrent vulvar and perianal itching, ulcers, and pain, leading to a diagnosis of genital herpes. In May 2022, the patient was referred to the hematology department due to elevated globulin levels and right inguinal lymphadenopathy. A complete blood count showed hemoglobin of 101 g/L (NR, 110–150 g/L), with no plasma cells detected in the peripheral blood smear. Biochemical tests indicated the following: total protein, 112.8 g/L (NR, 60–85 g/L); albumin, 37.1 g/L (NR, 40–55 g/L); globulin, 75.7 g/L (NR, 20–40 g/L); and beta-2-microglobulin, 4.4 mg/L (NR, 1–3 mg/L). Immunoglobulin levels were as follows: IgG, 67.4 g/L (NR, 8.6–17.4 g/L); IgA, 0.41 g/L (NR, 1.0–4.2 g/L); and IgM, 8.47 g/L (NR, 0.5–2.8 g/L). ANA profiling showed positive ANA (1:320), positive dsDNA (1:10), anti-SSA >400 RU/ml (NR, 0–20 RU/ml), and anti-SSB 101.01 RU/ml (NR, 0–20 RU/ml). Both EBV DNA and CMV DNA were negative. Lactate dehydrogenase, serum calcium, renal function, troponin I, and brain natriuretic peptide were all within normal limits. Serum protein electrophoresis revealed an M-protein accounting for 42% (absolute value: 47.38 g/L), and subsequent serum immunofixation electrophoresis confirmed the presence of an IgG-λ monoclonal protein. Urinalysis electrophoresis indicated positive M-protein (15.2%), and urine immunofixation electrophoresis further confirmed IgG-λ positivity. Serum free light chains (sFLC) were quantified as 26.9 mg/L (kappa) and 837.5 mg/L (lambda), with a λ:κ ratio of 31.13. Bone marrow smears showed hypercellularity with a notable increase in plasma cells (11%) (**Figure 2A**). Flow cytometry of bone marrow identified monoclonal abnormal plasma cells (3.71% of nucleated cells) expressing CD138, CD38, CD19, and cλ (cytoplasmic lambda), with negative CD56 expression (**Figures 2C, D**). Bone marrow biopsy revealed a significant increase in plasma cells (approximately 30% of nucleated cells), suggestive of plasma cell disorder. Immunohistochemical staining showed CD138(+), CD38(+), Igκ(-), Igλ(+), CD56(-), and Ki-67(+, <1%). FISH analysis was negative for 1q21 amplification, del 17p13, del 13/13q-, and IgH rearrangements. Chromosomal analysis revealed a normal karyotype (46, XX). Cardiac ultrasound showed no abnormalities. Before proceeding to excisional biopsy, an infectious work-up (HIV, EBV, CMV, tuberculosis PCR, syphilis serology) was performed and yielded negative results. Autoimmune-related reactive hyperplasia was considered as a differential, and to rule out lymphoma, the patient underwent excisional biopsy of the right inguinal lymph node on May 27, 2022. Histopathological analysis indicated that most of the lymph node architecture was preserved, with open sinuses, focal structural disruption, notable follicular hyperplasia, and focal granulomatous inflammation with necrosis (**Figure 2B**). Immunohistochemical staining showed CD20(+) and BCL-6(+) in follicular areas (Ki-67+, ~80%); CD3(+) and BCL-2(+) in interfollicular areas (Ki-67+, ~20%); and plasma cells positive for CD38 and CD138, with minority kappa light chain expression and predominant lambda light chain expression (indicating lambda light chain restriction) (**Figures 2E, F**). IgG was positive, while IgG4 was negative. Special staining showed negative acid-fast stain results, and PCR for TB-DNA was also negative. The pathological diagnosis was lymphoid hyperplasia with necrotizing granulomatous inflammation; combined with the patient’s medical history and immunohistochemical findings, this was suggestive of autoimmune disease progression accompanied by plasma cell dyscrasias.

**Figure 2:**
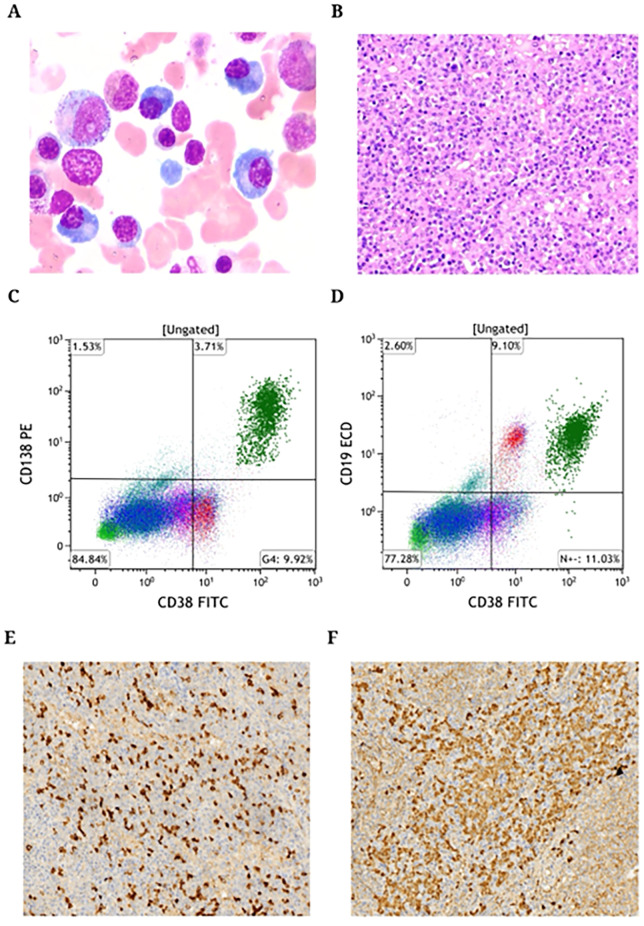
**(A)** Bone marrow smears revealed increased plasma cells and rouleaux formation of erythrocytes. **(B)** HE x 100 staining showed with open sinuses and disrupted structure in the lymph node some areas. **(C, D)** Flow cytometry revealed expression of CD38, CD138, and restricted expression of lambda. **(E, F)** IHC x 100 shows plasma cells in the lymph node with kappa **(E)** light chain expression noted in a minority and lambda **(F)** light chain expression predominantly, indicating lambda light chain restriction.

Multiple enlarged lymph nodes with mildly elevated glucose metabolism were identified by 18F-Fluorodeoxyglucose Positron Emission Tomography/Computed Tomography (PET/CT). Additionally, scattered inflammatory and fibrotic foci were observed in the right middle lobe and bilateral lower lobe basal segments, along with pulmonary bullae in the right middle lobe. Bilateral pleural thickening was also detected. (**Figure 3**).

**Figure 3:**
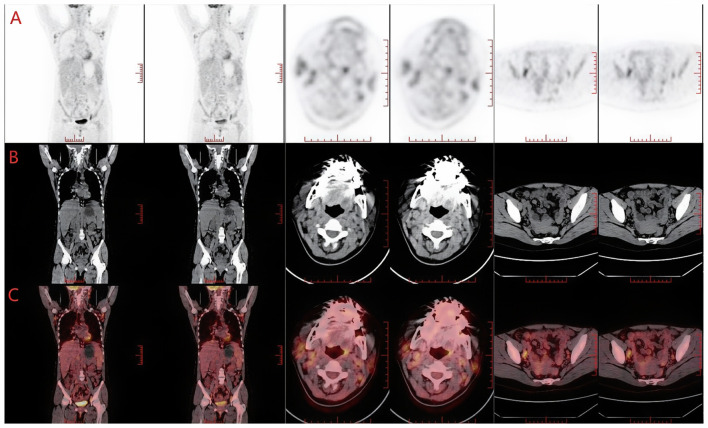
**(A)** No significant lytic bone lesions were detected by PET/CT. **(B, C)** Multiple enlarged lymph nodes were identified in the bilateral preauricular, parotid, submandibular, submental, cervical, axillary, iliac, and inguinal regions, as well as in the left parapharyngeal space, mediastinum, periaortic area, mesentery, presacral area, and perirectal region. Some lymph nodes showed fusion, with the largest measuring approximately 2.0 × 1.2 cm and an SUVmax of 5.7 (mildly elevated glucose metabolism). Bilateral parotid glands were enlarged with increased density, particularly on the right side (SUVmax 4.3). Mild diffuse metabolic increase was observed in both central and peripheral bone marrow (SUVmax 4.3).

Given the patient’s recurrent infections, lymphadenopathy, and autoimmune disease, an immune deficiency disorder was suspected. Further analysis of lymphocyte subsets showed the following: CD3+ T cells, 10 × 10^6^/L (NR, 955–2860 × 10^6^/L), CD3+CD4+ T cells, 2 × 10^6^/L (NR, 550–1440 × 10^6^/L); CD3+CD8+ T cells, 3 × 10^6^/L (NR, 320–1250 × 10^6^/L); CD4:CD8 ratio, 0.65; CD19+ B cells, 1 × 10^6^/L (NR, 90–560 × 10^6^/L); and natural killer (NK) cells, 3 × 10^6^/L (NR, 150–1100 × 10^6^/L). Whole-exome sequencing (WES) of peripheral blood identified a heterozygous mutation in the *PIK3CD* gene (c.1002C>G, p. Asn334Lys) (**Figure 4A**). Sanger sequencing validation of the patient’s mother confirmed no variation in exon 8 of the *PIK3CD* gene (**Figure 4B**), while Sanger sequencing of the patient’s son detected the same missense mutation (c.1002C>G, p. Asn334Lys) in exon 8 of the *PIK3CD* gene (**Figure 4C**), with no clinical symptoms observed in the son. Unfortunately, the patient’s father refused genetic testing.

**Figure 4:**
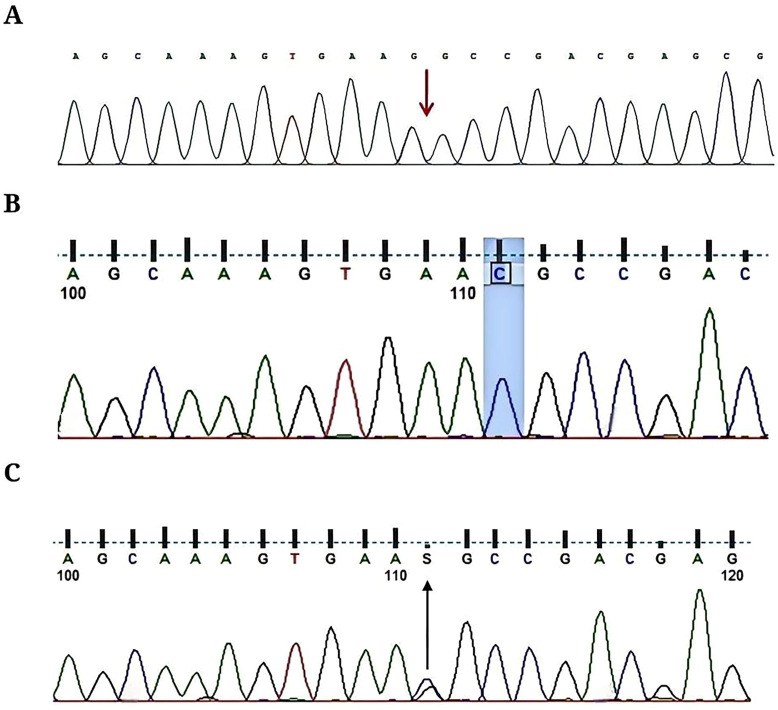
**(A)** A heterozygous mutation in the *PIK3CD* gene (c.1002C>G, p.Asn334Lys) was identified by WES. (Red arrow indicates the mutation site). **(B)** No missense mutation in exon 8 of the *PIK3CD* gene was detected in the patient’s mother via Sanger sequencing validation. **(C)** The same heterozygous mutation in the *PIK3CD* gene (c.1002C>G, p.Asn334Lys) in exon 8 was confirmed in the patient’s son by Sanger sequencing (Black arrow indicates the mutation site).

The patient was advised to consider sirolimus, immunoglobulin replacement, or allogeneic HSCT, but she declined due to financial constraints and opted for prophylactic antibiotics and antivirals (trimethoprim-sulfamethoxazole and valacyclovir). She consented to genetic testing of her 30-month-old son. Shortly after diagnosis, the patient was lost to follow-up; the last contact attempt (February 25, 2026) was unsuccessful. No further clinical data are available. The overall timeline of the patient's medical history and treatment is presented in **Figure 5**.

**Figure 5:**
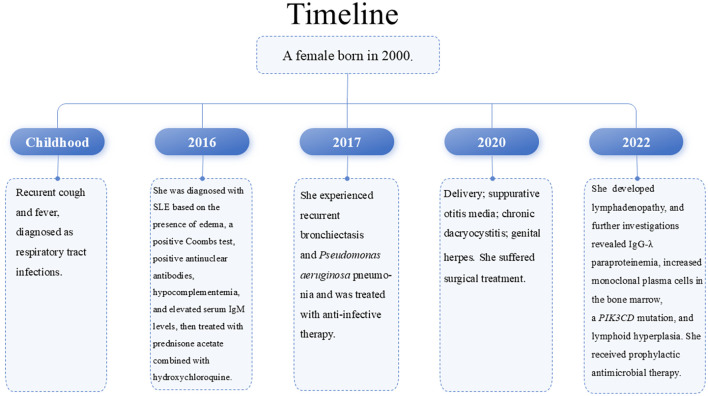
Case report timeline. SLE, systemic lupus erythematosus.

## Discussion

We report a case of APDS1 caused by a rare *PIK3CD* mutation (c.1002C>G, p. Asn334Lys) that coexists with SMM. To our knowledge, this is the first documented association between APDS1 and SMM. This finding significantly expands the phenotypic spectrum of tumor susceptibility in APDS1 and offers new insights into the role of PI3K signaling in plasma cell disease.

### Mechanistic considerations

Pathogenic variants in PIK3CD and PIK3R1 result in hyperactivation of the PI3K/AKT/mTOR signaling pathway in immune cells, particularly T and B lymphocytes, causing reduced B-, NK-, and T-cell numbers and impaired lymphocyte function (12). Meanwhile, increased activation of PI3Kδ in APDS predisposes to malignancy, and ovarian dysgerminoma, non-Hodgkin’s lymphoma (13), acute myeloid leukemia, basal cell carcinoma, Hodgkin’s lymphoma (14) have been reported. Furthermore, lymphoma, particularly B-cell lymphoma, is the most common malignancy in APDS, likely owing to PI3K pathway dysregulation in B cells, dysfunction of follicular helper T cells, impaired cytotoxicity of CD8+ T cells and NK cells, and defective regulatory T-cell activity (15). In MM, the PI3K/AKT/mTOR signaling pathway is also frequently dysregulated. Several myeloma growth factors (e.g., IL-6) exert their effects through this pathway, and PI3K/AKT/mTOR inhibitors can suppress MM tumor growth (16). Chronic inflammation, impaired immune surveillance, and sustained B-cell activation in APDS1 may collectively create a permissive environment for the emergence of clonal plasma cells. However, direct causality cannot be established from a single case; our report proposes a hypothesis-generating association that warrants further investigation.

### Anemia and CRAB criteria

SMM, a precursor condition of MM, is defined by 10%-60% clonal plasma cells in the bone marrow and/or serum monoclonal protein ≥30 g/L or urinary monoclonal protein ≥500 mg/24 h, in the absence of amyloidosis or myeloma-defining events (MDEs). MDEs include the CRAB features (Hypercalcemia, Renal insufficiency, Anemia attributable to myeloma (hemoglobin <100g/L or >20 g/L below the lower limit of normal, and Lytic bone lesions) and the SLiM criteria (clonal bone marrow plasma cell infiltration ≥60%, involved-to-uninvolved sFLC ratio ≥100, two or more focal lesions on MRI (each ≥5 mm in size)) (17). SMM typically presents in older adults (median age >60 years) and is rare under the age of 40, making its occurrence in this 22−year−old patient an unusual and noteworthy feature. The patient had mild anemia (hemoglobin 101 g/L) but no hypercalcemia, renal impairment, lytic bone lesions or any SLiM criteria. The anemia is most likely attributable to chronic disease—chronic inflammation related to APDS1 and active autoimmune features (hypocomplementemia, positive anti-SSA/SSB)—rather than a myeloma-defining event. Thus, the diagnosis of SMM remains appropriate according to International Myeloma Working Group (IMWG) criteria.

### IgG SMM versus hyper IgM

A distinctive immunological feature of this case is the coexistence of polyclonal hyper IgM (8.97 g/L) and a monoclonal IgG-λ protein (47.38 g/L). Immunofixation confirmed the M-protein as IgG-λ, while the elevated IgM was polyclonal. APDS1 causes generalized B-cell activation and class-switching defects. We hypothesize that in this patient, a single plasma cell clone successfully underwent class switching to IgG and became malignant, whereas most other B cells continue to produce high levels of polyclonal IgM.

### Choosing therapy for this patient

Based on the Mayo 2018 20/2/20 model (bone marrow clonal plasma cell infiltration >20%, serum M-protein >2 g/dL, involved/uninvolved free light chain ratio >20), this patient meets all three criteria for high-risk SMM (18), with an estimated 44% risk of progression to symptomatic multiple myeloma within 2 years (19). Treatment of SMM remains controversial, with some experts supporting early intervention to prevent organ damage and improve outcomes, while others oppose it because non-curative therapy may disrupt sub-clonal balance and promote the expansion of more aggressive, drug-resistant subclones (20, 21). Sirolimus, an mTOR inhibitor that has been used in APDS, may confer clinical benefit by mitigating non-neoplastic lymphoproliferative disease and improving NK cell function, although its efficacy against cytopenias appears limited (22–24). Leniolisib, a PI3Kδ inhibitor, has demonstrated good tolerability, with a reduction in lymphadenopathy and an increased proportion of naïve B cells in a cohort of 31 patients with APDS (10). Prolonged administration of leniolisib has been associated with significant improvements in health-related quality of life, alongside persistent alterations in lymphocyte subsets and clinical symptoms (25). Allogeneic HSCT is a promising therapeutic option for patients with APDS and may provide a potentially curative approach for hematopoietic and immune-related manifestations. In one report, nine patients with APDS1 underwent HSCT, yielding a cumulative survival rate of 86.1% in a cohort spanning 30 years (26). Another study reported a 2-year overall survival rate of 86% and a graft failure-free survival rate of 68% following allogeneic HSCT for APDS (27).

If the patient were still under follow-up today, leniolisib (approved in 2023) would be the preferred first-line therapy for APDS1. At the time of diagnosis (2022), only sirolimus, immunoglobulin replacement, and HSCT were available, all of which the patient declined. Despite high-risk SMM, we do not currently recommend initiating anti-myeloma therapy because of the patient’s underlying immunodeficiency and recurrent infections. If the patient were to progress to symptomatic MM, daratumumab-based regimens combined with leniolisib could be considered as a potential therapeutic option, although clinical evidence for this combination remains limited. Alternatively, a more aggressive approach would be to proceed with HSCT, as HSCT is theoretically capable of treating both APDS1 and MM. However, due to the patient’s financial constraints, she only receives prophylactic antibiotics and antivirals.

### Genetic testing of the asymptomatic son

The patient’s 30-month-old son carries the same *PIK3CD* mutation by Sanger sequencing but has no clinical symptoms. Genetic testing was performed at the family’s request after thorough counseling. Although no curative therapy is currently available, early diagnosis enables: (i) clinical surveillance and infection prevention, (ii) avoidance of live vaccines, (iii) timely consideration of targeted therapy if symptoms develop, and (iv) reproductive planning for the family. The family was informed of potential psychological implications and the option to defer testing until adulthood, and they chose immediate testing. We present this information not to encourage routine screening of asymptomatic minors but to illustrate individualized decision-making with full informed consent and ethical consideration.

### Strengths and limitations

Strengths of this report include the first description of APDS1 coexisting with SMM and the comprehensive clinical, immunological, pathological, and genetic workup. Limitations are: (i) a single case report; (ii) lack of functional validation of the PIK3CD variant; (iii) complete loss of the patient to follow-up after the initial diagnosis (last contact attempt on February 25, 2026 was unsuccessful), with no available data on disease course, treatment outcomes, or long-term progression; and (iv) the patient did not receive targeted therapy, so the dual effect on APDS1 and SMM could not be evaluated.

## Conclusion

Patients with childhood-onset recurrent infections, lymphadenopathy, autoimmunity, or growth retardation should be evaluated for APDS. Early WES and pedigree analysis facilitate timely diagnosis of APDS. Prompt treatment can improve patient prognosis. Treatment options for APDS include prophylactic antibiotics and antivirals, immunoglobulin replacement therapy, sirolimus, leniolisib, and allogeneic HSCT. APDS carries an increased risk of malignancies such as lymphoma, necessitating close monitoring. This case represents the first reported of APDS coexisting with SMM, expanding the disease spectrum and providing valuable insights for future studies.

## Data Availability

The original contributions presented in the study are included in the article/supplementary material, further inquiries can be directed to the corresponding author/s.
